# Characteristics stochastic analysis of long and narrow deep excavations under soil spatial variability

**DOI:** 10.1038/s41598-025-92948-z

**Published:** 2025-03-10

**Authors:** Shouhua Liu, Zhaoliang Wang, Yadong Chen, Guohua Fang, Yiding Zhao, Junsheng Yang, Huasheng Sun

**Affiliations:** 1 Architectural Engineering Institute, Huaiyin Institute of Technology, Huaian, 223001 Jiangsu China; 2 Huai’an Institute of Building Science Co., Ltd, Huai’an, 223004 Jiangsu China; 3 School of Civil Engineering, Yancheng Institute of Technology, Yancheng, 224051 Jiangsu China; 4 School of Civil Engineering, Central South University, Changsha, 410075 Hunan China

**Keywords:** Soil spatial variability, Deep excavation, Deformation mechanism, Reliability, Civil engineering, Mechanical engineering

## Abstract

The mechanical properties of soil, resulting from the weathering of rocks through physical and chemical processes, exhibit spatial variability. This variability introduces uncertainties in the design and characteristics of excavation projects. To address these uncertainties caused by soil spatial variability, safety factors are commonly used in excavation design. However, using the same safety factor for different indicators of soil spatial variability is illogical. Therefore, specialized research on the characteristics of deep excavations in the context of soil spatial variability is necessary, as it provides the theoretical basis for rational excavation design. In this study, we assumed that soil parameters follow a lognormal distribution, while spatial correlation adheres to a Gaussian function. We developed a random finite element algorithm for deep excavations, which incorporated Python programming and the ABAQUS computational platform. This algorithm was created within the framework of random field theory and Monte Carlo simulation. The results of our study indicate that, influenced by soil spatial variability, the lateral wall movements and ground surface settlements exhibit discrete distributions near the deterministic results. The maximum deformation of the excavation follows a normal distribution, while the pattern of ground surface settlements demonstrates diversity and chaotic characteristics. The extent to which soil spatial variability affects deep excavations is correlated with indicators of this variability. As the coefficient of soil spatial variability increases, the diversity and chaotic characteristics of ground surface settlements become more prominent. The locations of maximum ground surface settlement and maximum deformation becomes more scattered. Consequently, the probability of excavation failure increases, and the reliability index of the excavation decreases. In summary, soil spatial variability significantly impacts deformation prediction and safety control during the design and construction stages of deep excavations. Therefore, it is crucial to consider the influence of soil spatial variability when designing deep excavations, based on the variability indicators.

## Introduction

Deep excavation engineering^1–4^ is a high-risk project due to its comprehensiveness, complexity, and uncertainty. Design errors, improper construction, environmental factors, variability in soil parameters, insufficient monitoring, and poor management can all contribute to excavation accidents. Studies have revealed that design errors are the primary cause of accidents in deep excavation projects^5–7^. When an accident occurs, it not only endangers lives but also causes significant damage to the national economy and has negative effects on society. For instance, on April 20, 2004, a collapse occurred during the excavation of the Nicoll Highway section tunnel of the Singapore Metro Circle Line. This resulted in the complete collapse of a 100-meter retaining structure, leading to four fatalities and the disruption of the Nicoll Expressway. The incident caused extensive damage to the city’s infrastructure. On November 15, 2008, a collapse occurred at Xianghu Metro Station in Hangzhou, resulting in 21 deaths and a direct economic loss of 49.62 million. Furthermore, on May 11, 2017, a soil collapse took place during the deep excavation of a subway station in Shenzhen, resulting in three deaths, one injury, and a direct economic loss of 3.45 million. Therefore, it is crucial to adopt a scientifical and rational design approach to prevent accidents in deep excavations.

Soil, as a weathering product of rocks that undergo physical and chemical processes, exhibits spatial variability in its mechanical properties^8^. This variability introduces uncertainty in deep excavation design plans. Designers typically rely on safety factors recommended by codes^9–11^ to address the uncertainty caused by soil spatial variability. However, research by Duncan^12^ has revealed that using the same safety factors for designing deep excavations, while considering different indicators of soil spatial variability, is illogical. Consequently, accounting for soil spatial variability in a rational manner poses a significant challenge for excavation engineers. Currently, many scholars have used random field theory to research the effects of soil spatial variability on slopes^13–18^, tunnels^19–24^, and other engineering projects^21,25–32^, resulting in significant scientific advancements. Recently, researchers have begun studying the stability and deformation characteristics of deep excavations in the context of soil spatial variability. Lin Jun^33^ simulated soil spatial variability using a non-stationary lognormal random field based on CPTU test data. They analyzed the effects of the variation coefficient and fluctuation range of soil mechanical parameters on the stability of excavations, concluding that the influence of soil spatial variability on excavations should not be overlooked. Goh et al.^34^ investigated the influence of soil spatial variability on the basal heave stability of excavations using the reliability index method. Luo et al.^10,35,36^ examined the impact of soil spatial variability through the equivalent variance technique and proposed a simplified two-dimensional calculation method for assessing the basal heave stability of excavations. Sert et al.^11^, considering the influence of vertical spatial variability in sandy soil layers, studied how variations in the internal friction angle affect the deformation characteristics of cantilever deep excavations using the random finite element method. Gholampour and Johari^37^ extended the application of random fields to the reliability analysis of excavation support structures in unsaturated soils, providing a comprehensive analysis of how soil spatial variability affects excavation reliability. Based on random field theory, Yi Shun et al.^38^ and Li et al.^27^ conducted a detailed numerical analysis of the impact of soil spatial variability on the deformation characteristics of excavations.

In conclusion, researchers have studied the effects of soil spatial variability on excavation deformation and stability, demonstrating that the influence of soil spatial variability on excavations cannot be disregarded^11,27,33,34,37–43^. However, limited research has been conducted on the impact of soil spatial variability specifically on long and narrow deep excavations, which have a plan aspect ratio greater than 10 and a profile aspect ratio greater than 2^8,44^. As urban underground space utilization continues to develop, numerous long and narrow deep excavation projects are expected in the future. Therefore, understanding the deformation characteristics of these excavations under soil spatial variability is crucial for rational excavation design.

In light of this, we have developed a random finite element algorithm that accounts for the influence of soil spatial variability. This algorithm is based on random field theory and the Monte Carlo method. We assume that the geotechnical parameters follow a lognormal distribution, with spatial correlation modeled by a Gaussian function. To implement this algorithm, we utilize a combination of Python programming and the ABAQUS calculation platform. To investigate the influence of soil spatial variability on the deformation characteristics and reliability of long and narrow deep excavations, we have established multiple sets of random numerical analysis models. These models vary in terms of the coefficient of variation and fluctuation range, which serve as our research parameters. By studying these models, we can reveal the engineering implications of soil spatial variability. Overall, our work highlights the importance of considering soil spatial variability in the design and analysis of long and narrow deep excavations.

## A random finite element algorithm for deep excavations

### Method for simulating soil spatial variability

The lognormal stationary random field *H*(*x*, *y*) is employed to describe the spatial variability of soil. In this context, x and y denote the horizontal and vertical coordinates, respectively. To simulate the lognormal stationary random field of soil parameters, the midpoint discretization method based on Cholesky decomposition is utilized. The grid center coordinates for the random field of soil parameters are (*x*_*i*_, *y*_*i*_), where *i* ranges from 1 to *n*_*e*_, representing the total number of grid points. The autocorrelation function, *ρ*(*τ*_*x*_, *τ*_*y*_), employs a Gaussian-type (SQX) function. The autocorrelation matrix **C** is represented by the following equation:

$${\varvec{C}}=\left[ {\begin{array}{*{20}{l}} 1&{}& \cdots &{\rho \left( {\Delta {x_{1{n_e}}},\Delta {y_{1{n_e}}}} \right)} \\ {\rho \left( {\Delta {x_{12}},\Delta {y_{12}}} \right)}&1& \cdots &{\rho \left( {\Delta {x_{2{n_e}}},\Delta {y_{2{n_e}}}} \right)} \\ \vdots & \vdots & \ddots & \vdots \\ {\rho \left( {\Delta {x_{1{n_e}}},\Delta {y_{1{n_e}}}} \right)}&{}& \cdots &1 \end{array}} \right]$$

In the equation, Δ*x*_*ij*_ and Δ*y*_*ij*_ represent the relative distances in the horizontal and vertical directions from the centers of grids *i* and *j*, respectively. *ρ*(Δ*x*_*ij*_, Δ*y*_*ij*_) denotes the autocorrelation function.

The cross-correlation matrix **R** is described as:

$${\varvec{R}}={\left( {{\rho _{k,l}}} \right)_{m \times m}}$$

The dimension of the random field *H*^*G*^(*x*, *y*) is (*n*_*e*_×*m*)×*N*_*p*_. Its characteristics are determined by the number of grid divisions in the random field, the autocorrelation function, the fluctuation range, and the cross-correlation coefficient, all of which collectively influence the random field. To transform the random field *H*^*G*^(*x*,*y*) in space, the following equation can be used:

$$H_{{{X_i}}}^{{k,NG}}(x,y)=F_{i}^{{ - 1}}\left\{ {\Phi \left[ {H_{{{X_i}}}^{{k,G}}(x,y)} \right]} \right\},k=1,2, \ldots ,{N_p},i=1,2, \ldots ,m$$

In the equation, *F*^-1^_*i*_(·) represents the cumulative distribution function, while Φ(·) represents the standard normal distribution function.

Based on the obtained soil parameters from the random field, a logarithmic normal distribution of soil parameters is achieved in ABAQUS through coordinate mapping. This approach enables us to calculate the characteristics of excavations while considering the spatial variability of the soil.

### Development of random finite element algorithm for deep excavations

Based on the Python language and the ABAQUS platform, we have developed a random finite element algorithm for deep excavations by combining numerical analysis with random field theory. By utilizing the variability indices of soil parameters, this algorithm enables the discretization of the random field of soil parameters and provides the mechanical response of deep excavations under conditions of soil variability. The detailed process can be accomplished in four steps.

Step 1: Establish the excavation model and extract coordinate information. First, create the excavation model in ABAQUS and divide the grid. Extract the node information for each element from the generated INP file, and create separate files for the node and element information.

Step 2: Determine statistical parameters to generate a random field. Identify statistical parameters such as the mean, coefficient of variation, and cross-correlation coefficient for soil strength and deformation parameters. Select the Gaussian-type (SQX) autocorrelation function and define the fluctuation range. Develop a Python program to generate lognormal stationary random fields. Utilize the node and element information obtained in the first step to produce N sets of lognormal stationary random fields for elastic modulus, cohesion, and internal friction angle.

Step 3: Establish the excavation model, input soil parameters, and acquire the initial stress field in the soil. Use a Python program to implement the entire process of model establishment, parameter assignment, calculation, and generation of the initial INP file. Next, use a Python script to modify the strength and deformation parameters in the INP file and generate N INP files with random field parameters. Finally, submit batch calculation programs through ABAQUS COMMAND to obtain N initial stress fields.

Step 4: Batch stress equilibrium. In the Python script generated in Step 3, add field variables and their corresponding parameter values. Additionally, import the initial stress field obtained in Step 3 and modify the model keywords to achieve ground stress equilibrium and obtain the final INP file. Afterward, you can quickly generate N INP files with random field parameter information by modifying the strength parameters in this INP file using a Python script. Finally, submit batch calculation programs through ABAQUS COMMAND to obtain N result files.

### Verification of random finite element algorithm for deep excavations

**Fig. 1:**
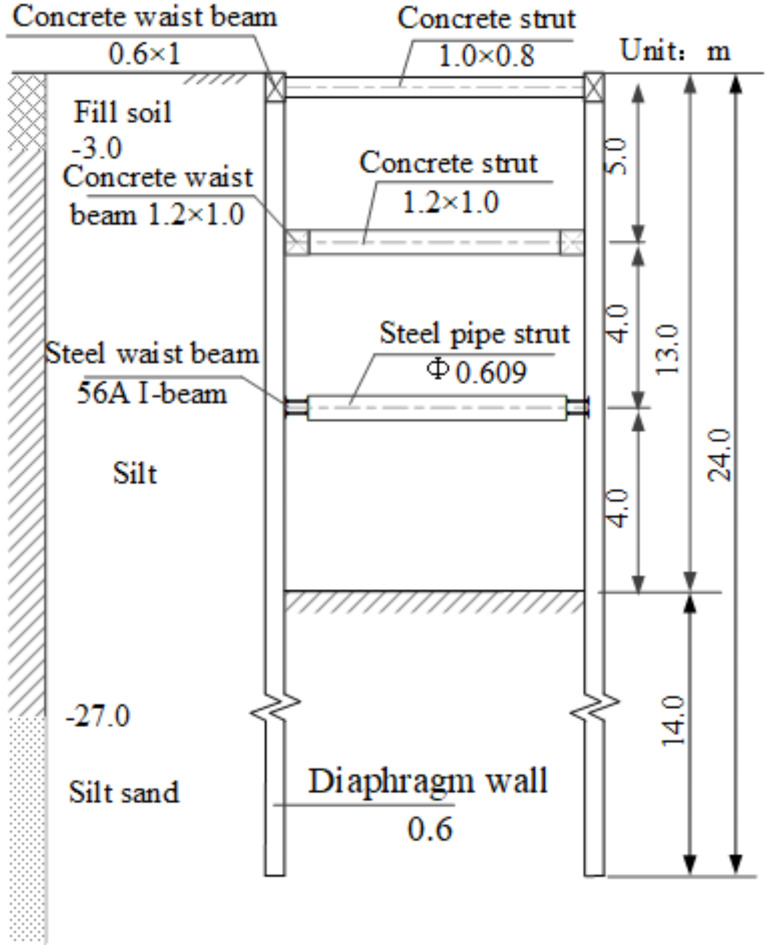
Cross-section of the supporting scheme.

**Fig. 2:**
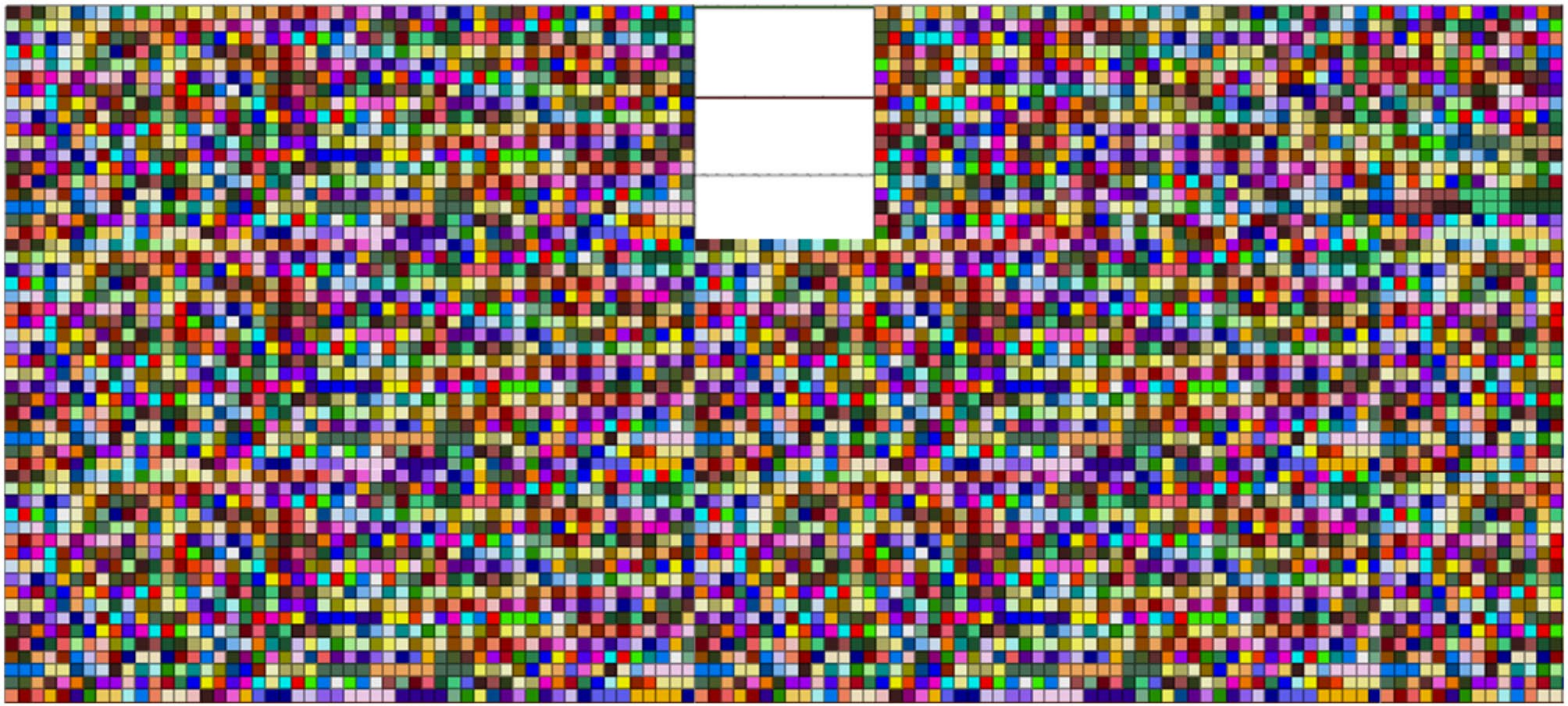
Soil property grid and support structure for a single Monte-Carlo simulation.

To validate the random finite element algorithm for deep foundation pits, the railway tunnel open-cut narrow and long deep foundation pit project in Guangzhou was selected as the analysis case. Detailed parameters of the support structure are shown in Fig. 1, while geological parameters are presented in Table 1^45^. Analysis files for the deep foundation pit were generated, and 1,000 Monte Carlo simulations were conducted. Figure 2 illustrates the soil property grid and support structure for a single Monte Carlo simulation.

**Table 1:** Statistical results of geological parameters^45^.

Soil layer	Statistical item	*γ*	*E*_s_	*c*	*φ*
		kN/m^3^	MPa	kPa	°
Fill soil	Count	10	10	10	10
	Maximum value	20.2	5.02	12	15.9
	Minimum value	17.7	2.86	4	9.5
	Mean value	19.5	3.9	7	12
	Standard deviation	0.195	0.507	0.77	1.08
	Coefficient of variation	0.01	0.13	0.11	0.09
Silt sand	Count	32	32	32	32
	Maximum value	25.2	42.6	8	42.5
	Minimum value	22.4	32.4	3	36.2
	Mean value	23	38.7	5	40
	Standard deviation	1.173	4.64	0.29	6
	Coefficient of variation	0.051	0.12	0.058	0.15
Silt	Count	314	201	32	32
	Maximum value	17.8	3.3	11.9	7.8
	Minimum value	14.1	1	2.5	2.1
	Mean value	15.9	2.1	7.8	4.1
	Standard deviation	0.69	0.67	2.7	1.6
	Coefficient of variation	0.044	0.336	0.355	0.371

*γ*: weight of soil; *E*_s_: compression modulus; *c*: cohesive forces; *φ*: internal friction angle.

**Fig. 3:**
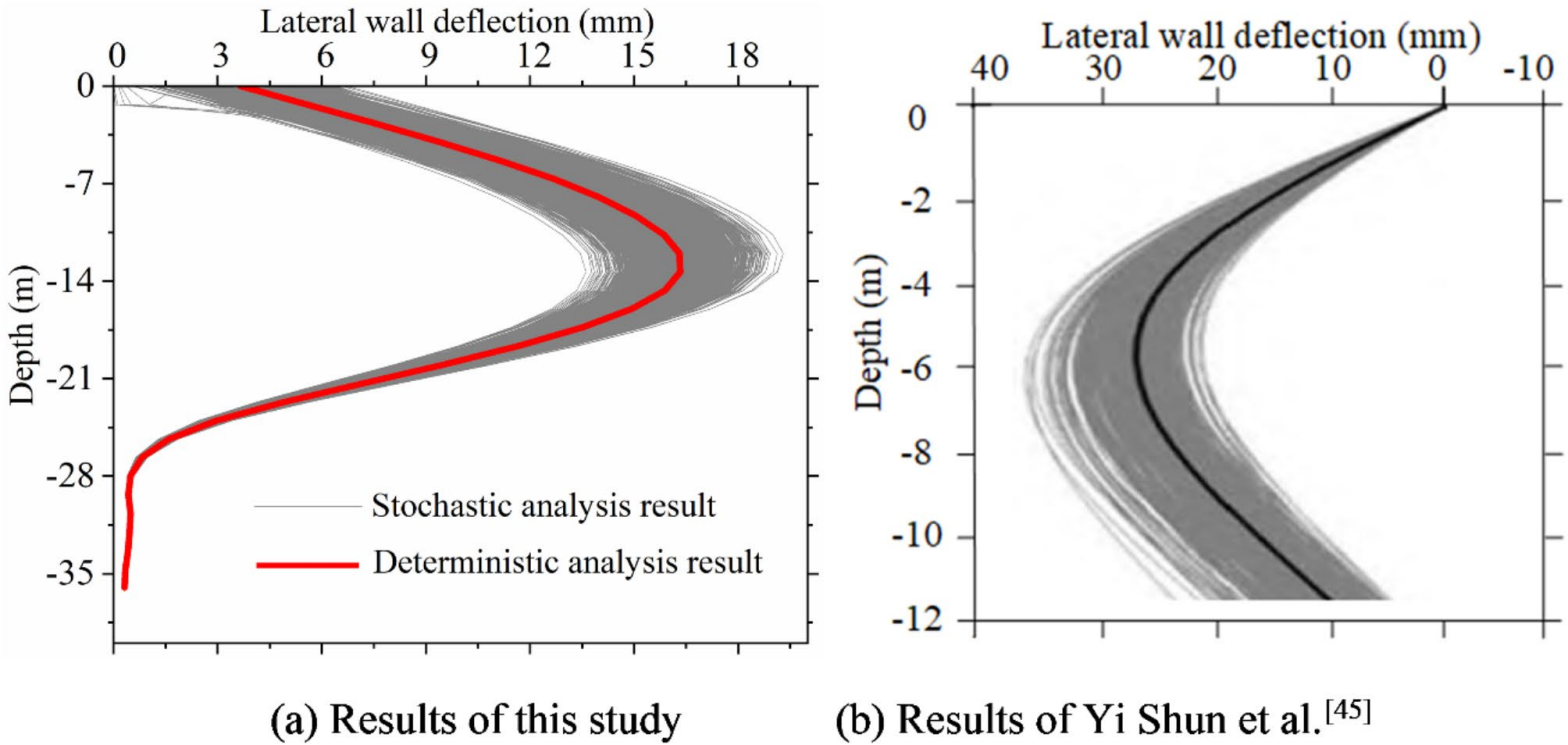
Comparison of lateral wall deflection.

**Fig. 4:**
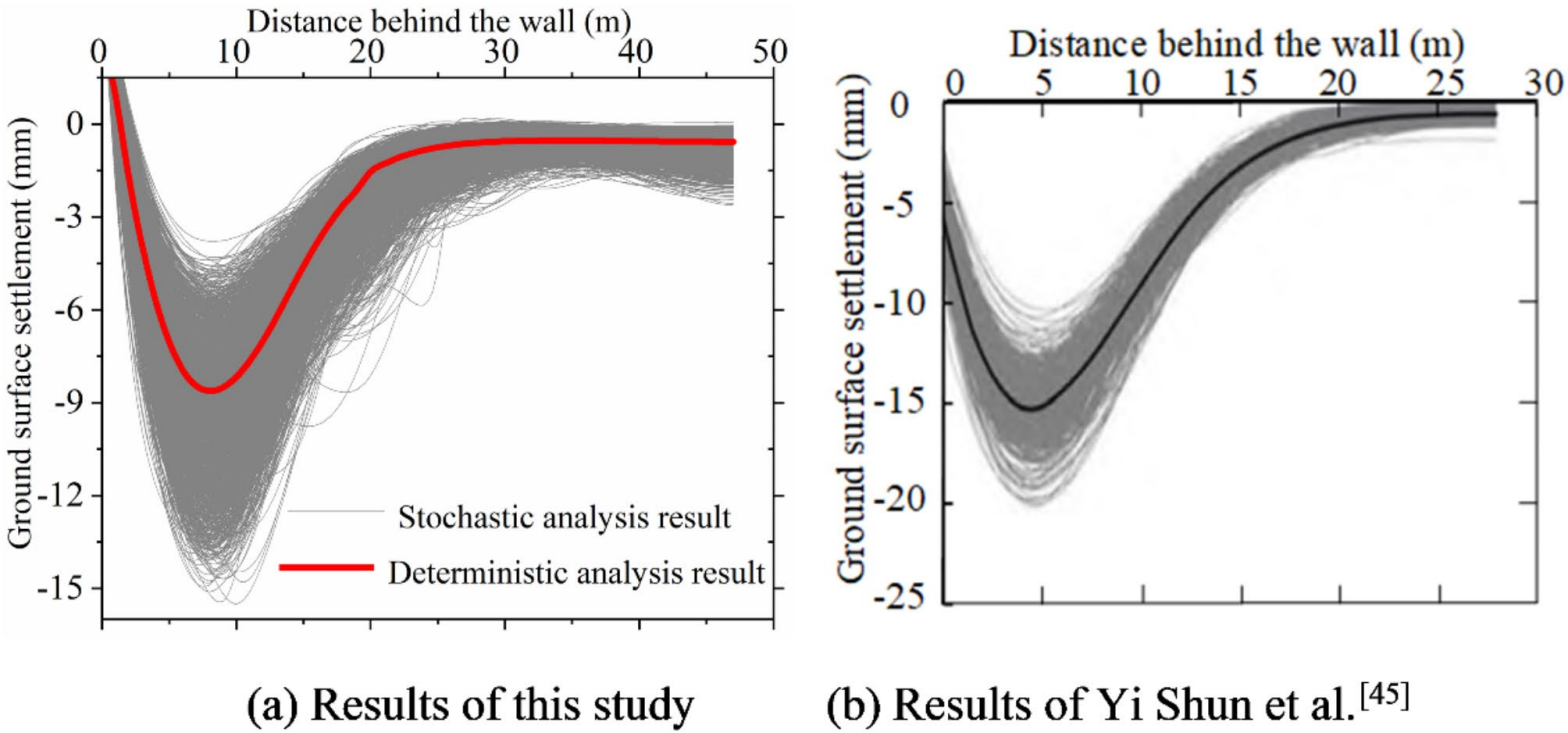
Comparison of ground surface settlement.

Organizing the results of 1,000 random finite element calculations and comparing them with the results of Yi Shun et al.^38^, it is evident from Figs. 3 and 4 that the lateral wall deflections and ground surface settlement curves of deep excavation under soil spatial variability align with Yi Shun et al.’s findings^38^. This validation confirms the effectiveness of the random finite element algorithm.

## Characteristics of long and narrow deep excavations

### Setting of working conditions

According to the research results, the study mainly focuses on the spatial variability of soil deformation parameters (Elastic modulus E) and strength parameters (Cohesion c and Internal friction angle φ). Through statistical analysis of published literature^15,18,21,46–50^, it was found that there is a significant difference in the fluctuation ranges of rock and soil in the horizontal and vertical directions. The horizontal fluctuation range is typically much larger than the vertical fluctuation range, with the horizontal range between 10 and 50 m and the vertical range between 0.5 and 5 m, exhibiting a coefficient of variation (COV) ranging from 0.1 to 0.5. Additionally, previous studies have indicated that the impact of horizontal fluctuation range variation on excavation pits is much smaller compared to that of the vertical fluctuation range^33,38^. To capture the influence of horizontal displacement fluctuation on excavation pits while controlling calculation time, the horizontal fluctuation range is set at 30 m. Furthermore, the coefficient of variation is set at 0.1, 0.2, 0.3, 0.4, and 0.5, while the vertical fluctuation range is set at 1 m, 2 m, 3 m, 4 m, and 5 m. This results in a total of 25 working conditions. For each working condition, 1000 Monte Carlo random finite element simulations are conducted, resulting in a total of 20,000 random models.

### Analysis of ground surface settlement

The ground surface settlement curve and maximum ground surface settlement are important indicators for assessing the impact of excavation construction on the surrounding environment.

**Fig. 5:**
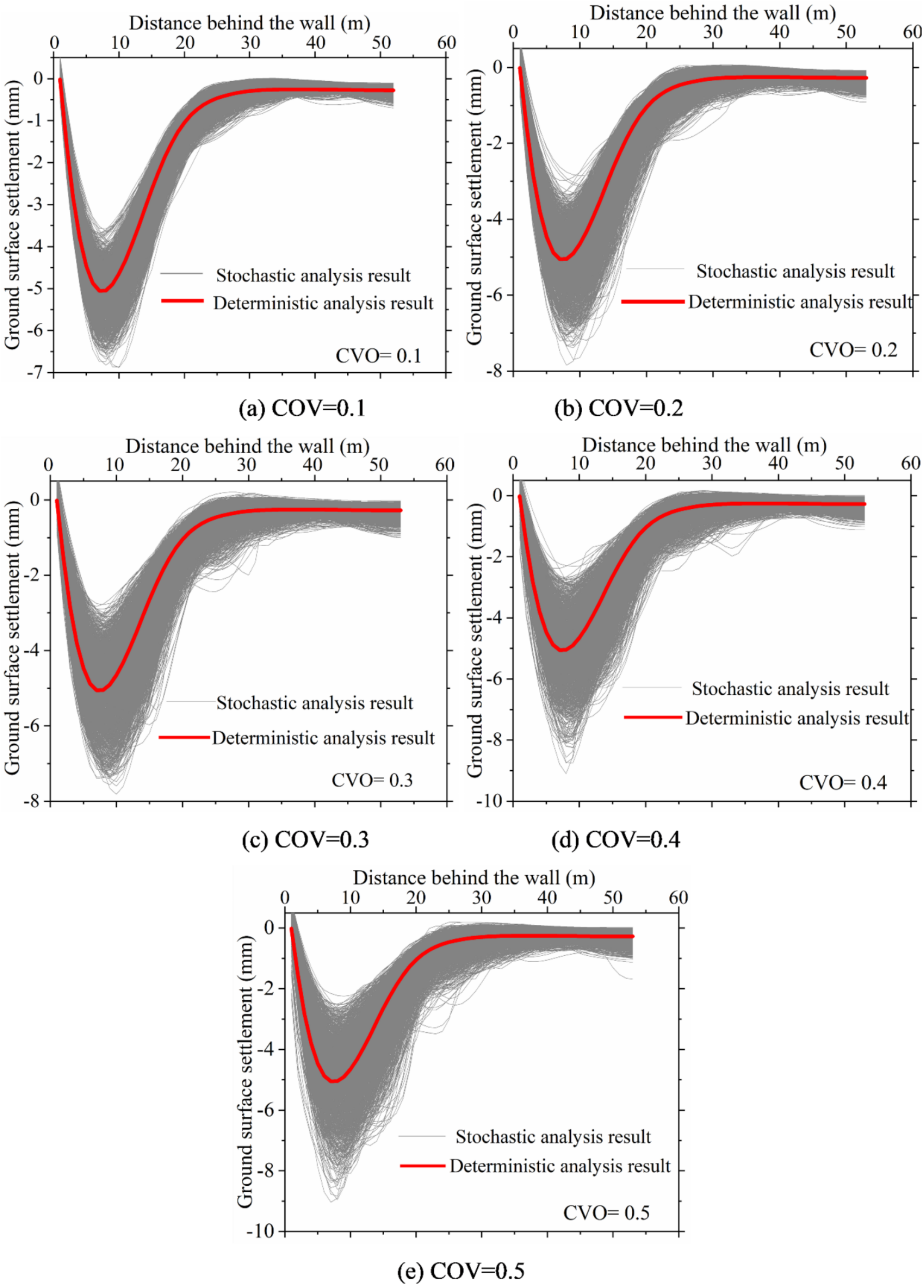
Ground surface settlement curves under different COV.

Figure 5 illustrates the ground surface settlement curves under different coefficients of variation. It can be observed that, due to soil spatial variability, the settlement curves exhibit a discrete distribution, fluctuating randomly above and below the deterministic results. This spatial variability in the soil leads to diverse and chaotic characteristics in the patterns of ground surface settlement curves, with these features becoming more pronounced as the coefficient of variation increases.

**Fig. 6:**
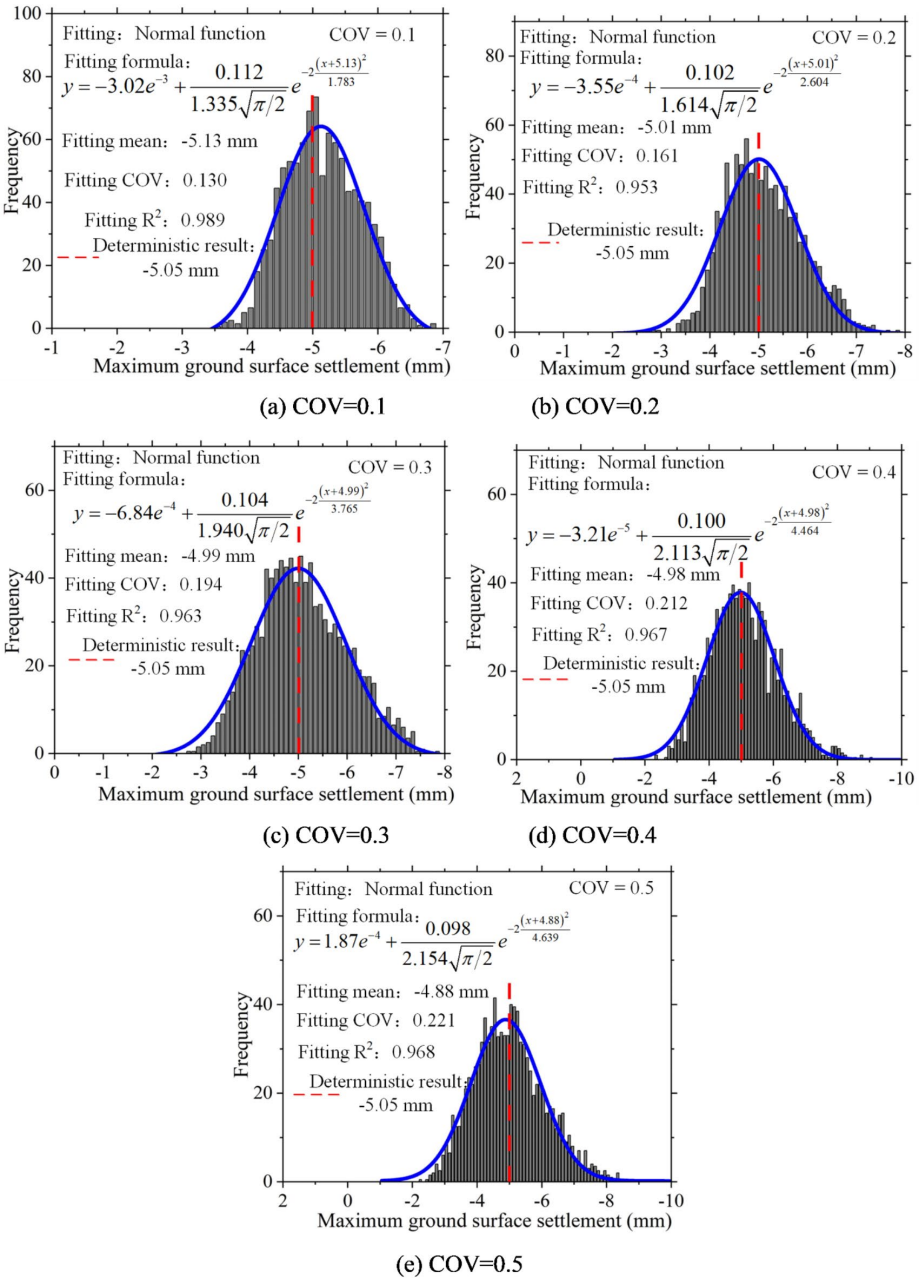
Frequency histogram of maximum ground surface settlement under different COV.

Statistical analysis was conducted on the maximum ground surface settlement, and a histogram of the frequency distribution of this maximum settlement was plotted. Figure 6 demonstrates that, in the presence of soil spatial variability, the maximum ground surface settlement exhibits a discrete distribution that follows a normal distribution pattern. Additionally, it is worth noting that as the coefficient of variation increases, the fitted coefficient of variation also increases, indicating greater dispersion in the distribution of maximum ground surface settlement.

**Fig. 7:**
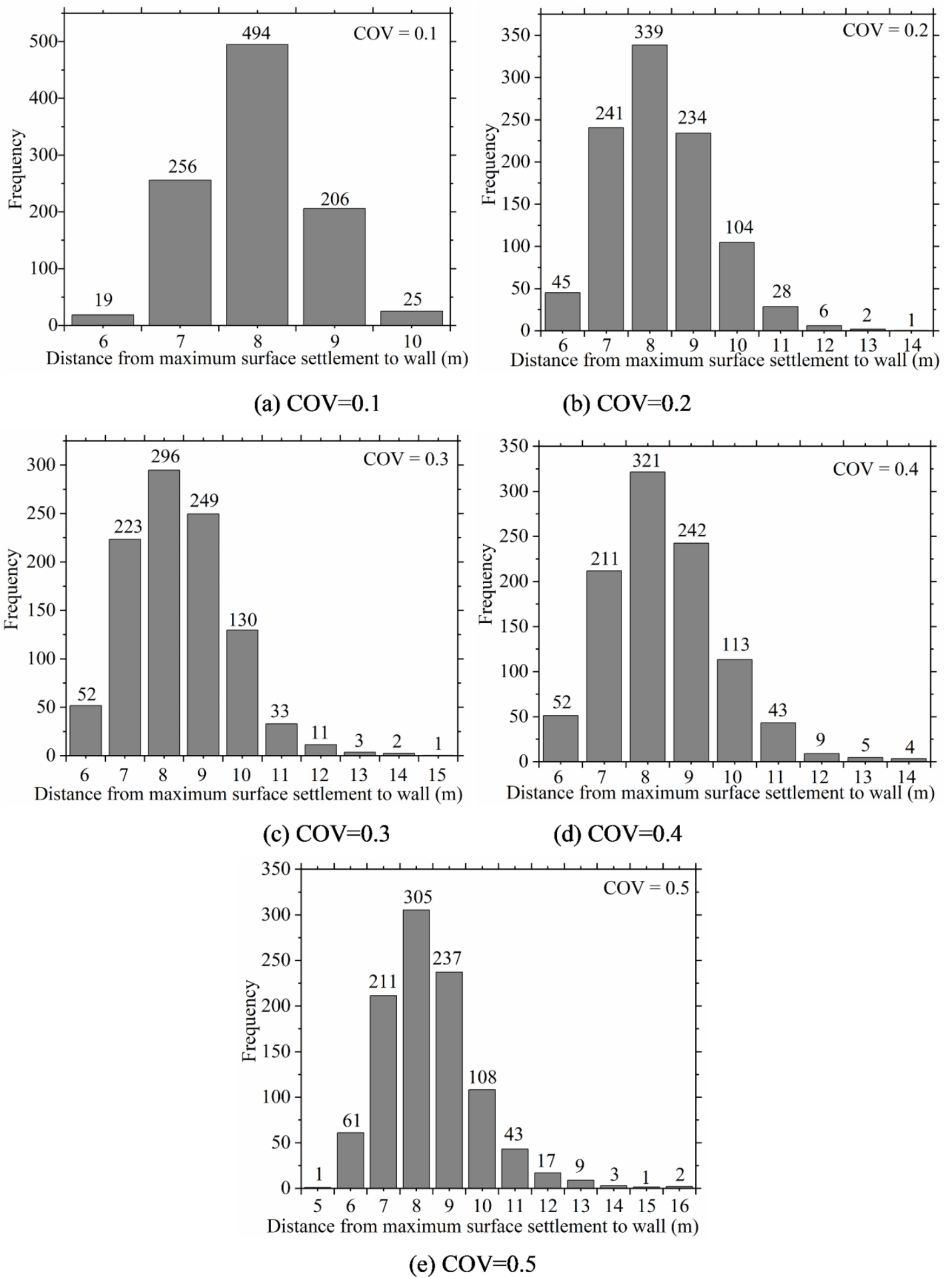
Frequency histogram of maximum ground surface settlement position under different COV.

The location of maximum ground surface settlement is an important indicator for assessing the impact of excavation on the surrounding environment. Therefore, the locations of maximum ground surface settlement were statistically analyzed to better understand the influence of soil spatial variability. The histogram in Fig. 7 presents the locations of maximum ground surface settlement. It reveals that, due to soil spatial variability, the location of maximum settlement is not fixed but rather falls within a certain range. Furthermore, as the coefficient of variation increases, the area of maximum settlement expands continuously. This suggests that excavation exerts a greater impact on the surrounding environment.

**Fig. 8:**
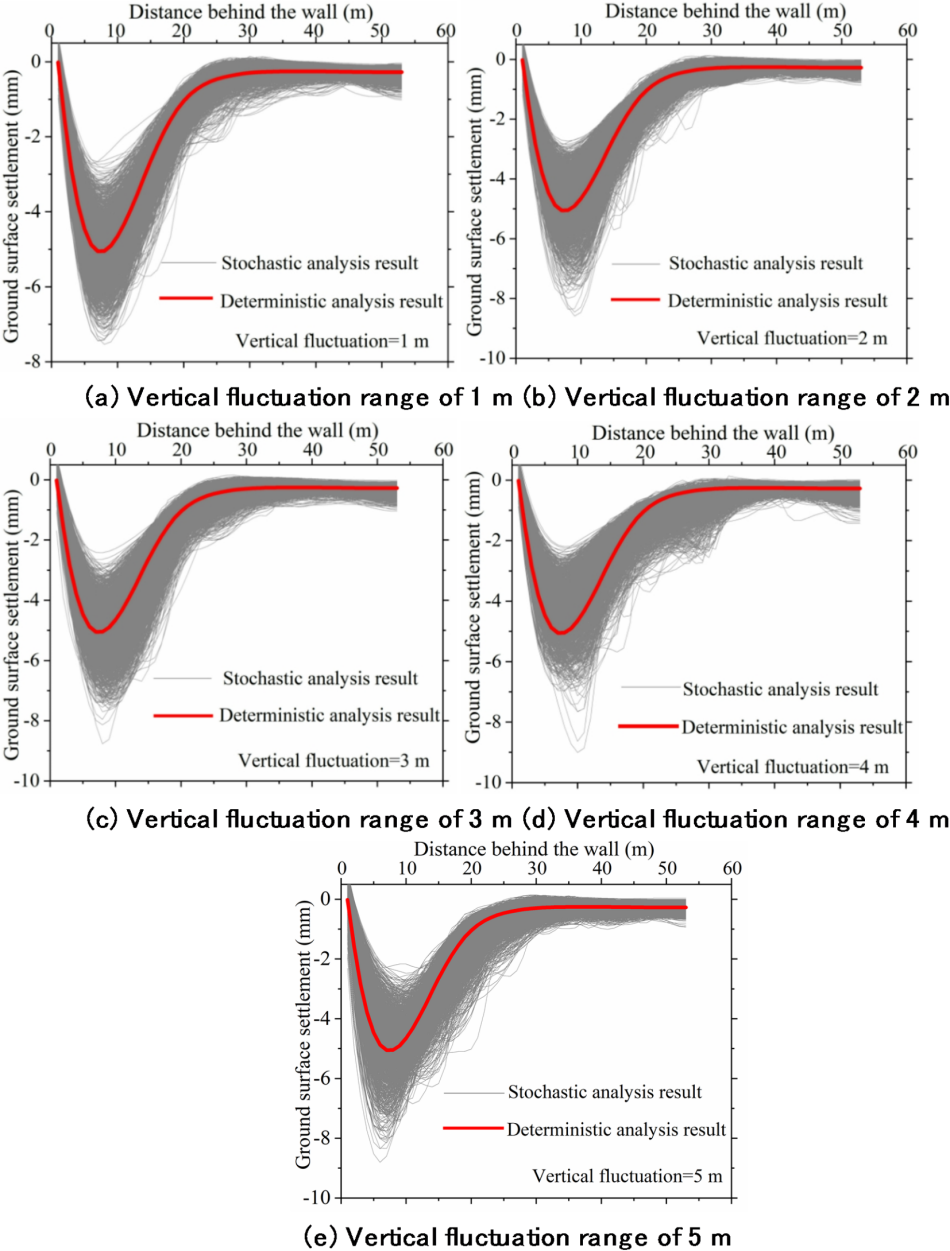
Ground surface settlement curves under different vertical fluctuation.

**Fig. 9:**
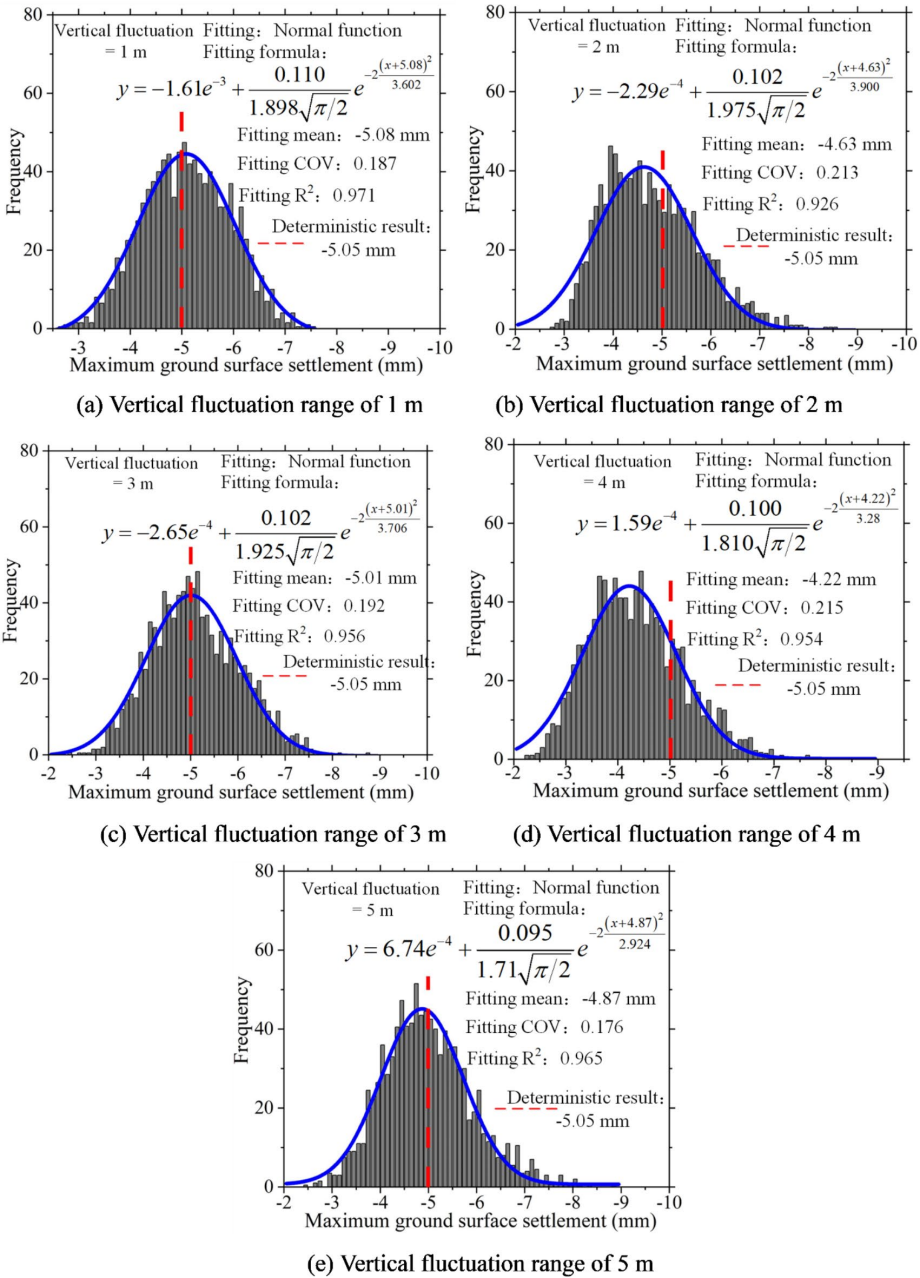
Frequency histogram of maximum ground surface settlement under different vertical fluctuation range.

**Fig. 10:**
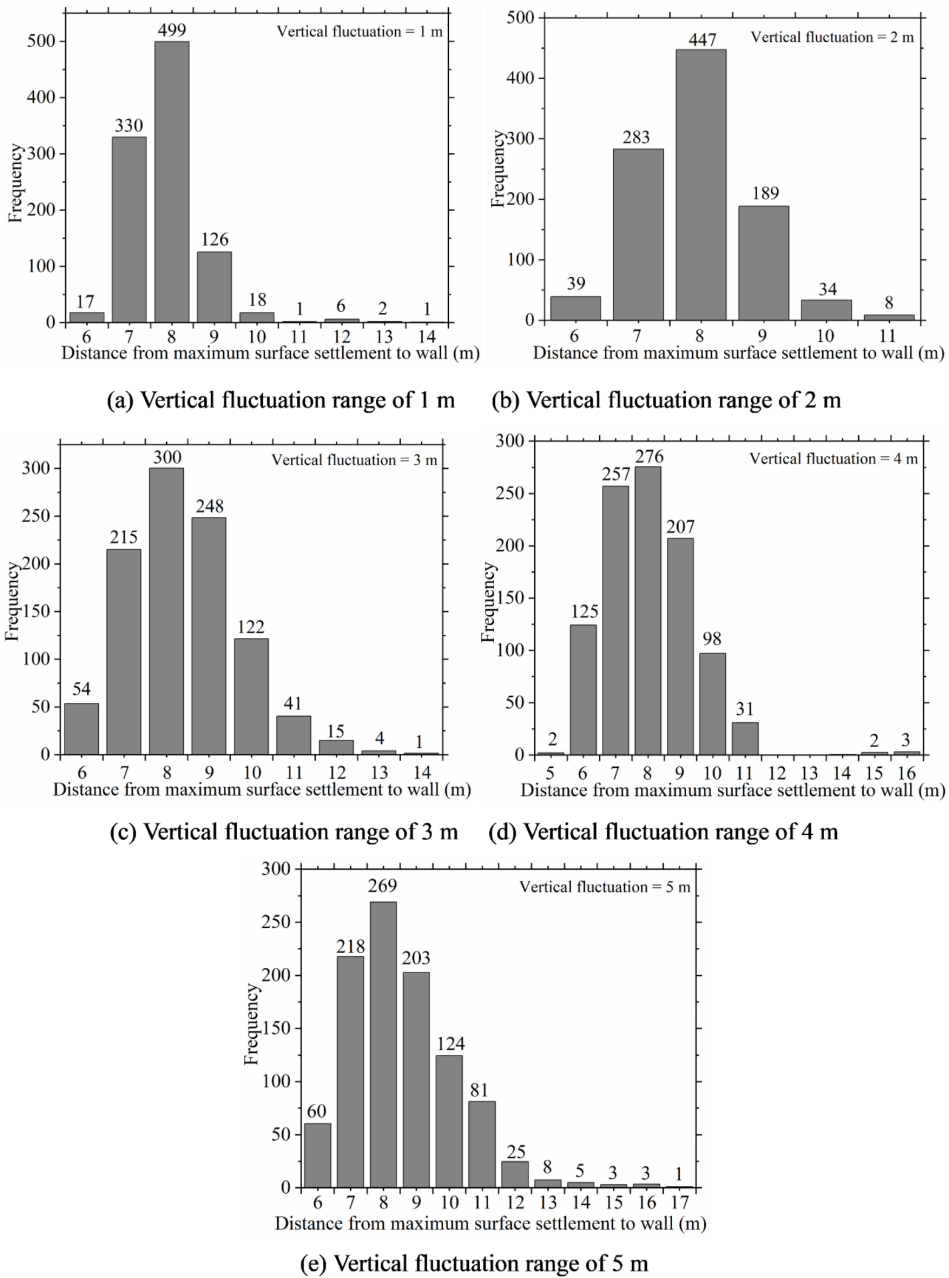
Frequency histogram of maximum ground surface settlement position under different vertical fluctuation range.

Figures 8, 9 and 10 provide a statistical analysis of ground surface settlement characteristics under various ranges of vertical fluctuation. It can be observed from the figures that the variations in the vertical fluctuation range on ground surface settlement characteristics lack consistency in their impact. However, changes in the vertical fluctuation range significantly affect ground surface settlement. In the future, to fully consider the impact of soil spatial variability, special attention should be paid to the vertical fluctuation range of the soil during the investigation and design phases of excavations.

### Analysis of lateral wall deflection

**Fig. 11:**
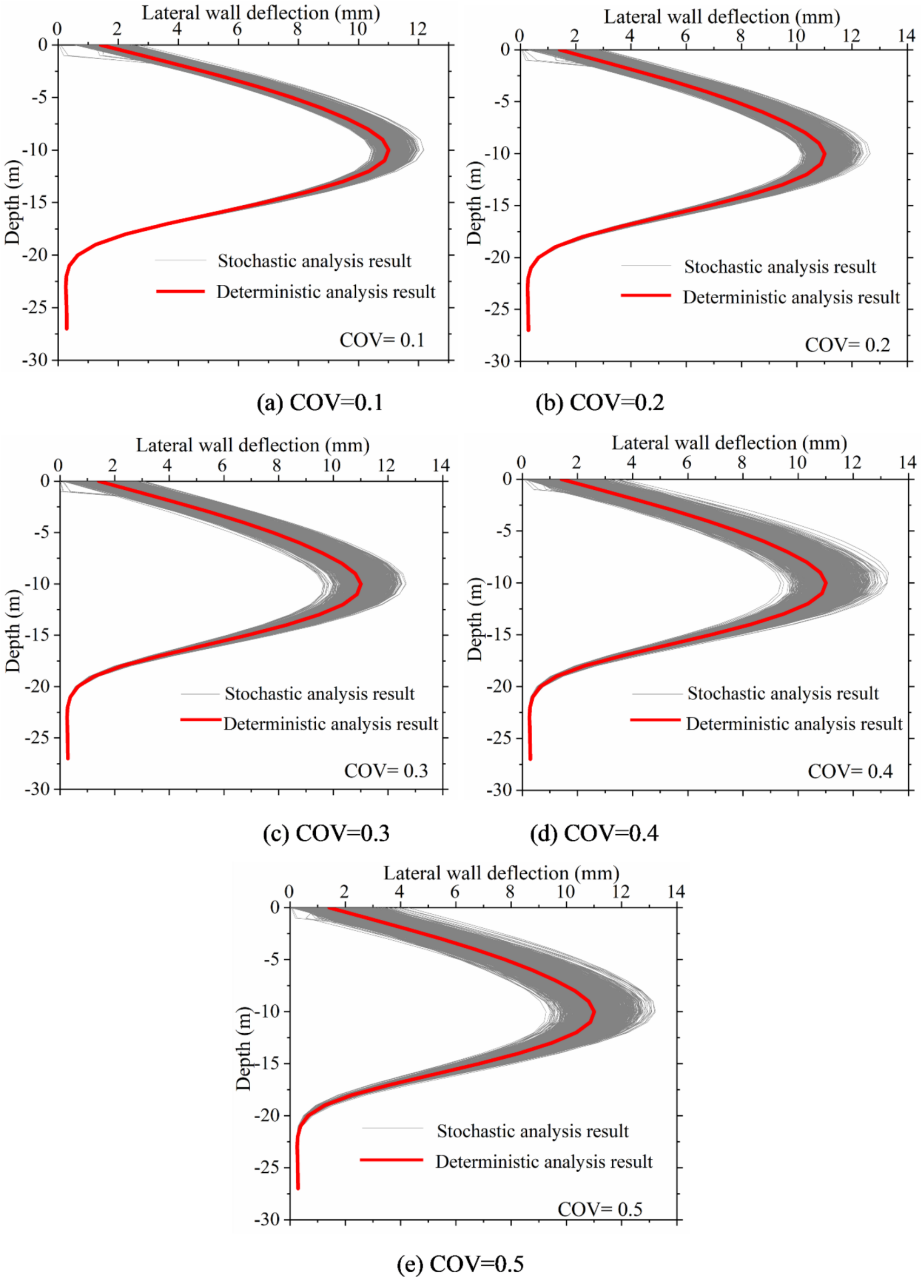
Lateral wall deflection curves under different COV.

The impact of excavation on the surrounding environment is characterized by ground surface settlement, while the safety of the excavation itself is primarily assessed through the deflections of the retaining structure. Figure 11 displays the lateral wall deflection curves under different coefficients of variation. It can be observed that lateral wall deflection curves exhibit a discrete distribution due to the influence of soil spatial variability, fluctuating randomly around deterministic results. Moreover, as the coefficient of variation increases, the degree of dispersion in the distribution of the lateral wall deflection curves also rises. Despite this variability, the lateral wall deflection curves still demonstrate a “compound” deformation mode under soil spatial variability, consistent with the mode obtained from deterministic analysis.

**Fig. 12:**
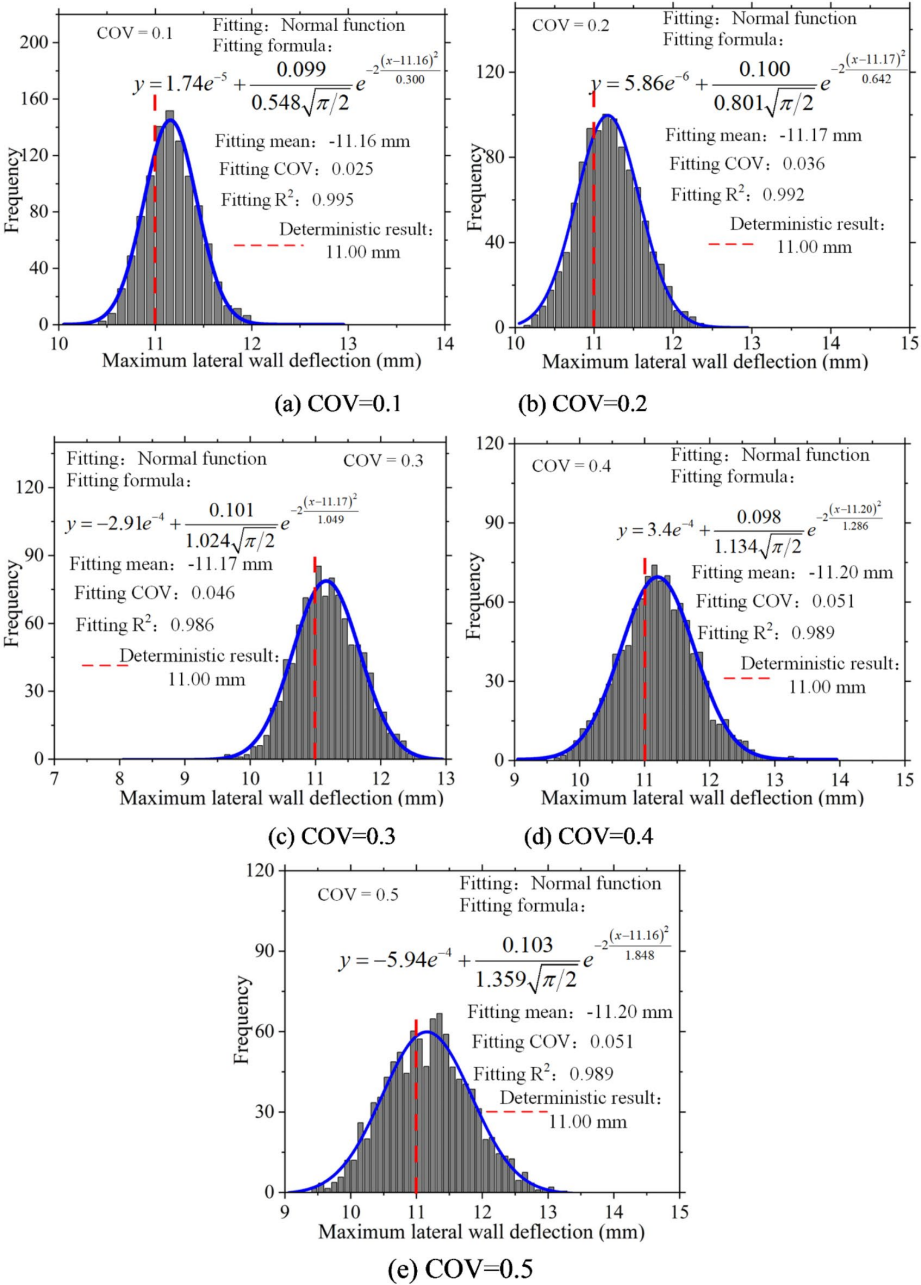
Frequency histogram of maximum lateral wall deflection.

Statistical analysis was conducted on the maximum lateral wall deflection, and a histogram of its frequency distribution was plotted. Figure 12 clearly illustrates that, in the presence of soil spatial variability, the maximum lateral wall deflection exhibits a discrete distribution and follows a normal distribution pattern. Furthermore, as the coefficient of variation increases, the fitted coefficient of variation also rises, indicating greater dispersion in the distribution of maximum lateral wall deflection.

The wall deformation mode and the maximum lateral wall deflection are important indicators for assessing excavation characteristics. Additionally, the location of maximum lateral wall deflection is a crucial reference point for excavation. Table 2 presents a statistical analysis of the location of maximum lateral wall deflection (*H*_hm_) under different analysis conditions. The table shows that, for various analysis conditions, there is a greater than 95% probability that the maximum lateral wall deflection occurs at the same location. This indicates that soil spatial variability has little influence on the location of maximum lateral wall deflection.

**Table 2:** Statistical analysis of the maximum lateral wall deflection position.

Working conditions	H_hm_ = − 8 m Counts	H_hm_ = − 9 m Counts	H_hm_ = − 10 m Counts	H_hm_ = − 11 m Counts	H_hm_ = − 10 m Probability
N-1	0	0	1000	0	100.0%
N-2	0	0	1000	0	100.0%
N-3	0	2	998	0	99.8%
N-4	0	7	993	0	99.3%
N-5	0	0	986	14	98.6%
F-1	0	4	995	1	99.5%
F-2	0	5	989	6	98.9%
F-3	8	8	978	14	97.8%
F-4	10	30	946	14	94.6%
F-5	10	22	939	29	93.9%
E-1	0	0	1000	0	100.0%
E-2	0	0	998	2	99.8%
E-3	0	0	987	13	98.7%
E-4	5	0	960	35	96.0%
E-5	0	5	958	37	95.8%
Y-1	0	0	1000	0	100.0%
Y-2	0	0	999	1	99.9%
Y-3	0	1	998	1	99.8%
Y-4	2	5	993	0	99.3%
Y-5	3	6	991	0	99.1%

**Fig. 13:**
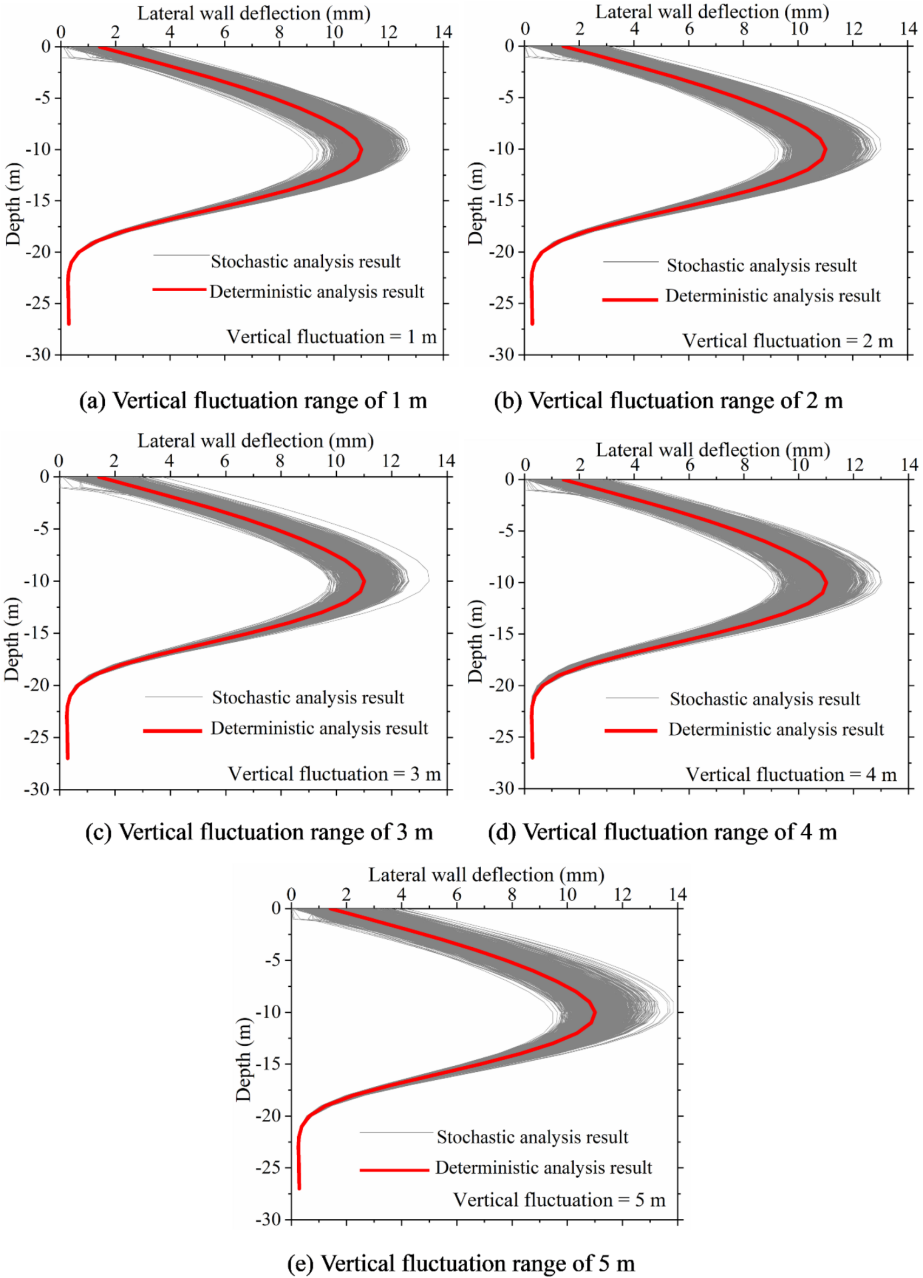
Lateral wall deflection curves under different vertical fluctuation range.

**Fig. 14:**
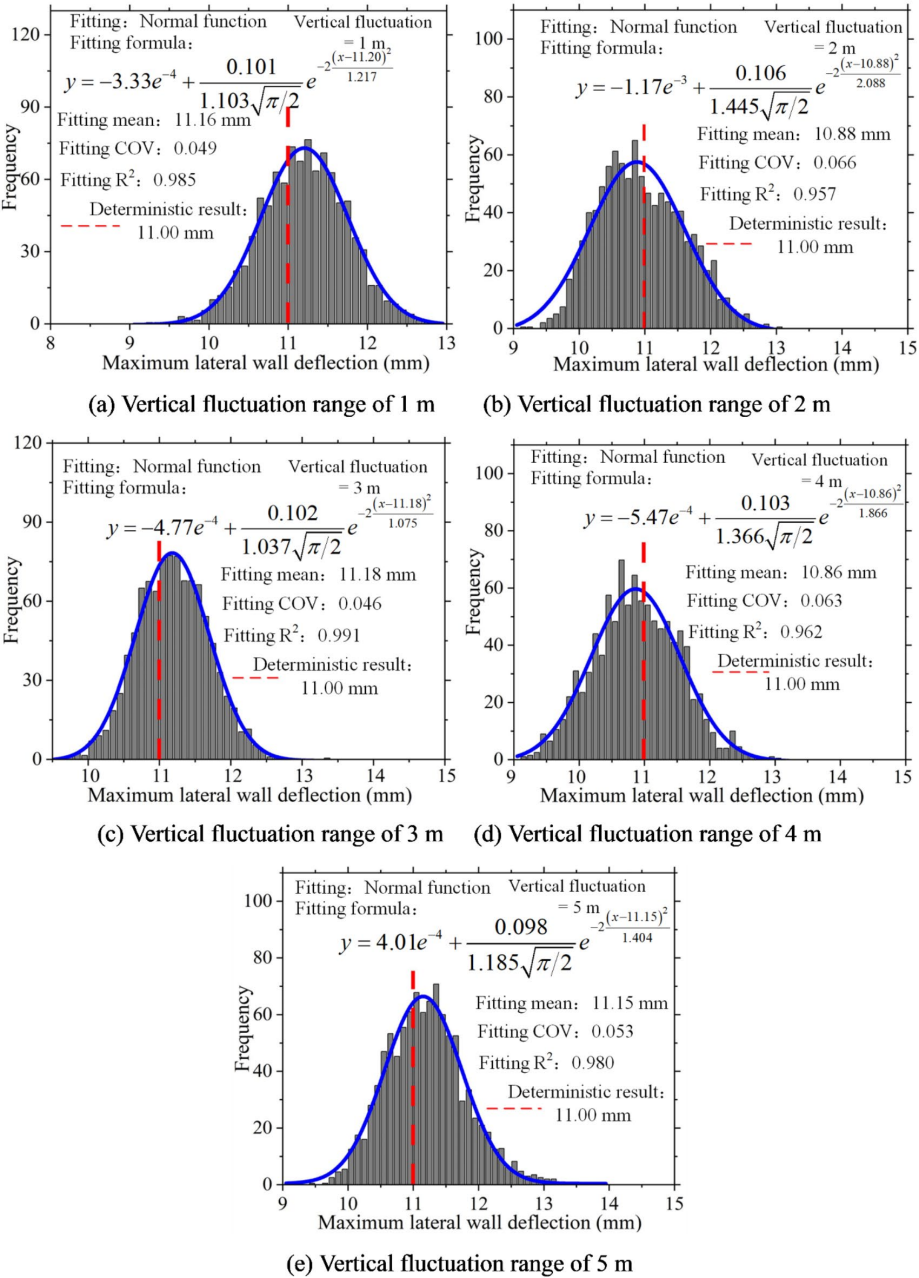
Frequency histogram of maximum lateral wall deflections.

Figures 13 and 14 present a statistical analysis of lateral wall deflection characteristics under various vertical fluctuation ranges. The figures indicate that the fluctuation range influences the characteristics of lateral wall deflections lack consistency. However, changes in the vertical fluctuation range significantly affect lateral wall deflections. In the future, to fully consider the impact of soil spatial variability, special attention should be paid to the vertical fluctuation range of the soil during the investigation and design phases of excavations.

In summary, under soil spatial variability, the ground surface settlement curves and lateral wall deflection curves exhibit discrete distributions, with the location of maximum ground surface settlement also showing a discrete distribution. Additionally, the characteristics of excavations influenced by soil spatial variability are related to variability indicators. Soil spatial variability affects the prediction of stratum deformation during the excavations design phase, posing challenges to safety control measures for the excavation.

## Reliability analysis of long and narrow deep excavations

Due to the various factors influencing excavation deformation, expressing excavation deformation as an explicit function of soil parameters is challenging. Although ABAQUS software can calculate excavation deformation in random models, the high computational cost associated with these random models makes it difficult to determine the failure probability (*P*_*f*_) and reliability index (*β*) of the excavation. Nevertheless, results from 1,000 random simulations demonstrate that both the maximum lateral wall deflection (*D*) and maximum ground surface settlement (*V*) of the excavation follow a normal distribution. To simplify the calculations, we can assume that variables *D* and *V* are independent, allowing us to use the Monte Carlo simulation method to determine the failure probability (*P*_*f*_) and reliability index (*β*) of the excavation.

After implementing 100,000 Monte Carlo simulations using MATLAB programming to calculate the failure probability *P*_*f*_ and reliability index *β*, the functions for the maximum lateral wall deflection (*D*) and maximum ground surface settlement (*V*) are as follows.

$$\begin{aligned} Z_{1} = & D_{u} - D \\ Z_{2} = & V_{u} - V \\ \end{aligned}$$

In the equation, *D*_*u*_ represents the warning value for the maximum lateral wall deflection, set at 30 mm^51^. Likewise, *V*_*u*_ indicates the warning value for the maximum ground surface settlement, set at 25 mm^51^.

**Fig. 15:**
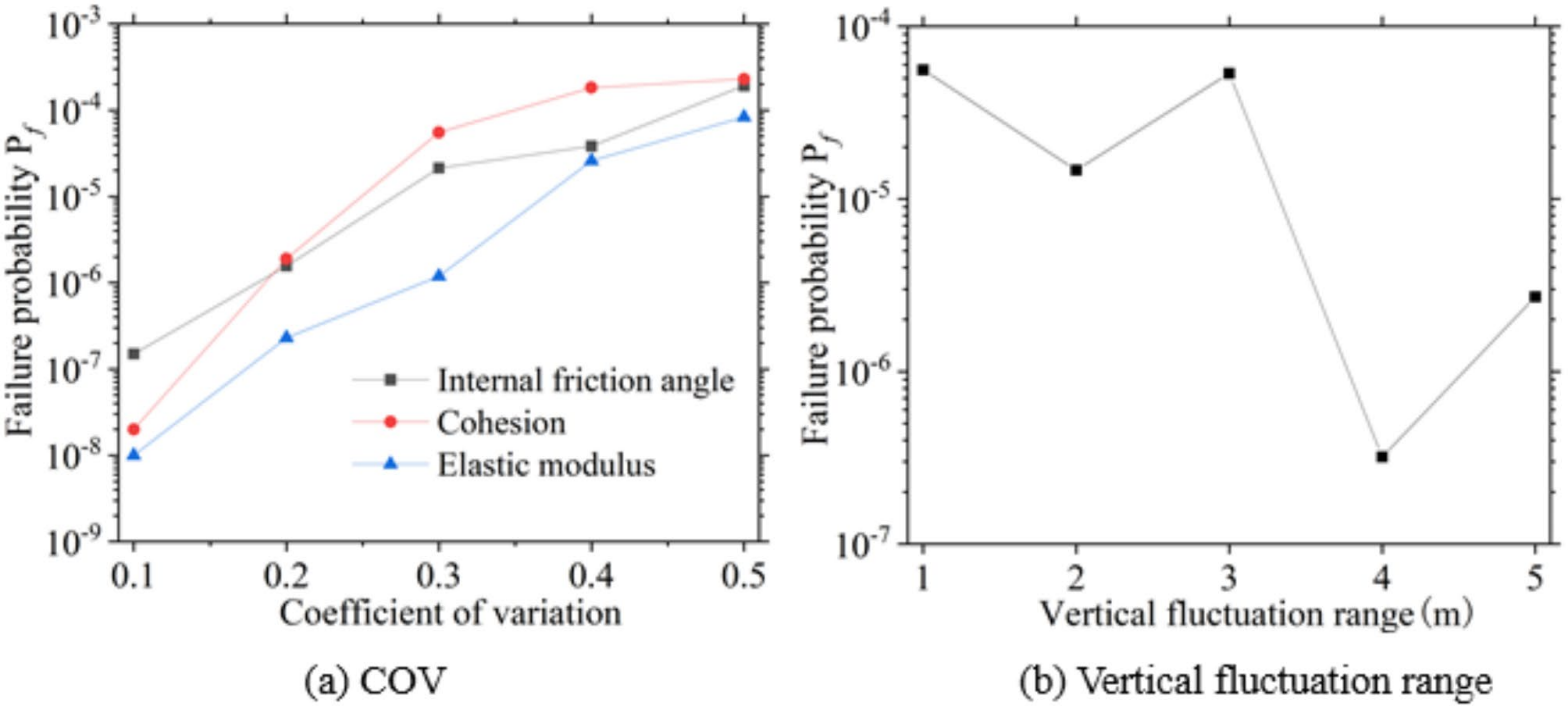
Excavation failure probability under soil spatial variability.

**Fig. 16:**
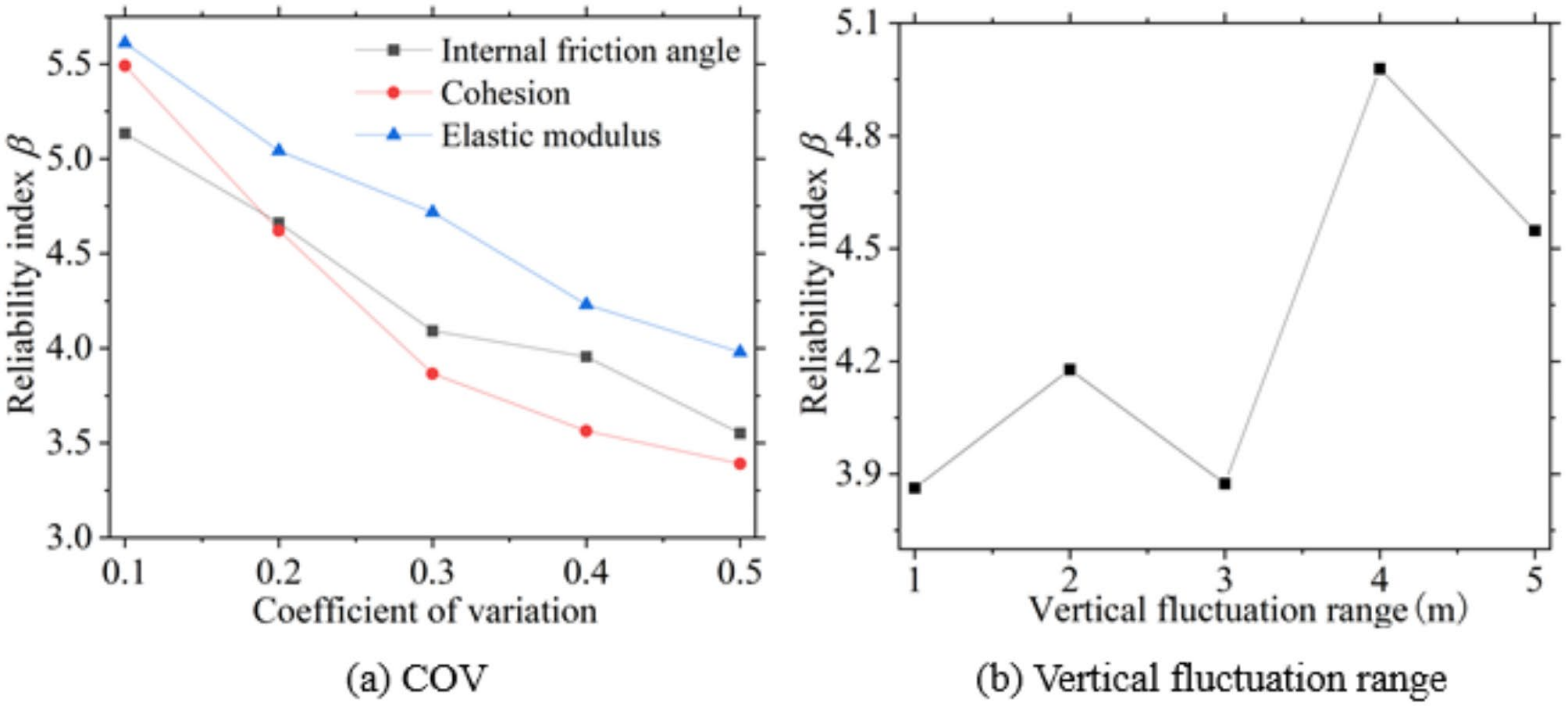
Excavation reliability index under soil spatial variability.

Figures 15 and 16 illustrate the failure probability and reliability index of excavations under various coefficients of variation and ranges of fluctuation. These indices are influenced by the spatial variability of the soil; higher coefficients of variation lead to an increased failure probability and a decreased reliability index for the excavations. Additionally, the fluctuation ranges also have an unpredictable impact on the failure probability and reliability index, resulting in varying outcomes for these indices.

## Conclusions

In this paper, a random finite element algorithm for deep excavations has been developed, taking into account soil spatial variability. Multiple sets of long and narrow deep excavation random numerical analysis models have been established, incorporating the coefficient of variation and fluctuation range. This approach enables us to study the impact of soil spatial variability on long and narrow deep excavations. Based on our analysis, we have drawn the following conclusions:

1. Assuming that the soil mechanical parameters follow a lognormal distribution and that spatial correlation follows a Gaussian function, a random finite element algorithm for deep excavations was developed. This algorithm accounts for the influence of soil spatial variability and was created using Python programming and the ABAQUS computational platform. It is based on random field theory and Monte Carlo principles.
2. Under the influence of soil spatial variability, the curves of lateral wall deflection and ground surface settlement are discretely distributed around the deterministic results. This distribution exhibits diverse and chaotic characteristics in ground surface settlement. Both the maximum ground surface settlement and the maximum lateral wall deflection follow a normal distribution, although the location of the maximum ground surface settlement is discrete. As the coefficient of variation in soil parameters increases, the diversity and chaotic characteristics of ground surface settlement become more pronounced. Additionally, the location of the maximum ground surface settlement becomes increasingly discrete, as do the maximum ground surface settlement and the maximum lateral wall deflection. The excavation characteristics are also influenced by the fluctuation range of soil parameters, although the relationship between them is not highly regular.
3. The spatial variability of soil has a substantial impact on the failure probability and reliability index of long and narrow deep excavations. As the coefficient of variation of soil parameters increases, the failure probability of excavations also increases, while the reliability index decreases. Additionally, the fluctuation range of soil parameters affects the reliability of excavations, although the relationship between these factors is not well understood.
4. The spatial variability of soil can significantly impact excavation characteristics and reliability. The extent of this influence is determined by the spatial variability index, which poses challenges for deformation prediction and safety control during the design and construction phases of deep excavations. Therefore, in the excavation design stage, the spatial variability of soil can be reasonably accounted for using the finite element algorithm developed in this paper.

## Data Availability

The datasets used and/or analysed during the current study available from the corresponding author on reasonable request.
